# Endolumenal Vacuum Therapy and Fistulojejunostomy in the Management of Sleeve Gastrectomy Staple Line Leaks

**DOI:** 10.1155/2018/2494069

**Published:** 2018-03-04

**Authors:** Kyle Szymanski, Estrellita Ontiveros, James S. Burdick, Daniel Davis, Steven G. Leeds

**Affiliations:** ^1^Division of Minimally Invasive Surgery, Baylor University Medical Center at Dallas, Dallas, TX 75246, USA; ^2^Department of Gastroenterology, Baylor University Medical Center at Dallas, Dallas, TX 75246, USA; ^3^Division of Bariatric Surgery, Baylor University Medical Center at Dallas, Dallas, TX 75246, USA

## Abstract

Laparoscopic sleeve gastrectomy (LSG) is the most common bariatric surgery performed for morbid obesity. Leaks of the vertical staple line can occur in up to 7% of cases and are difficult to manage. Endolumenal vacuum (EVAC) therapy and fistulojejunostomy (FJ) have separate documented uses to heal these complicated leaks. We aim to show the benefit of using EVAC with FJ in the treatment of LSG staple line leaks. The patient presented with an LSG chronic leak. EVAC therapy was initiated but failed to close the fistula after 101 days. EVAC therapy was abandoned, and FJ was performed to resolve the leak. Postoperatively, no leak was encountered requiring any additional procedures. Based on our findings, we conclude that EVAC therapy facilitates in resolving leaks that restore gastrointestinal continuity and maintain source control. It promotes healing and causes reperfusion of ischemic tissue and fistula cavity debridement.

## 1. Introduction

Laparoscopic sleeve gastrectomy (LSG) is used in the treatment of morbid obesity due to its perceived surgical simplicity and excellent outcomes [1]. A feared complication is a leak along the surgical staple line. These leaks have been reported in up to 7% of cases [2, 3]. Some current surgical interventions have been used to treat these leaks including Roux-en-Y fistulojejunostomy (FJ) [2, 3]. Our group has used endolumenal vacuum (EVAC) therapy to successfully heal leaks from sleeve gastrectomies [4]. We plan to discuss a case where FJ was used to treat a sleeve gastrectomy leak in conjunction with EVAC.

## 2. Case Presentation

We received Institutional Review Board approval for one patient that had a prolonged EVAC course for a chronic staple line fistula from SG, and EVAC was abandoned for surgical intervention with FJ. The EVAC therapy procedure used is described in depth in our previous publication [4]. In short, a nasogastric tube is used to deliver the negative pressure through the endosponge.

**Figure 1:**
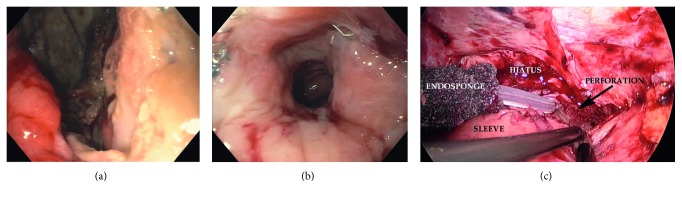
(a) View of the proximal sleeve perforation site prior to intervention. (b) View of the perforation after EVAC therapy. Cavity appears debrided, well perfused, and not infected. (c) Laparoscopic view of the hiatus at the time of FJ. Adhesions were taken down to see the proximal perforation site, and the Endo-SPONGE has been pulled through the perforation into the peritoneal cavity.

A 33-year-old female underwent an adjustable gastric band resulting in erosion, and three months later, she underwent SG. No endoscopic evaluation was done prior to proceeding with SG. She had a staple line leak (Figure 1(a)) on postoperative day 5 with associated septic shock. Laparotomy was performed for washout, omental patch, and a feeding jejunostomy tube. A stent was attempted, but migration resulted in stent removal, and EVAC therapy was initiated. The time from leak diagnosis to EVAC initiation was 84 days. Serial esophagogastroduodenoscopies with Endo-SPONGE removal failed to show resolution of the leak (Figure 1(b)). Laparoscopic FJ was performed 101 days following the initiation of EVAC (Figure 1(c)). She was discharged home 23 days after her surgery on a soft diet.

## 3. Discussion

The case presented here is an introduction to EVAC therapy and its effects on contaminated tissue during a gastrointestinal leak following bariatric surgery. The patient that underwent EVAC therapy for 101 days and was taken for FJ after being considered an EVAC failure revealed some interesting characteristics about EVAC therapy. Following that procedure, the patient had an upper gastrointestinal exam that demonstrated no leak and was started on a diet. We are proposing that the use of EVAC therapy can be used in conjunction with FJ creation and facilitate healing.

We are projecting that the chronic nature of the fistula [1] in this patient would have had a poorer outcome if EVAC was not used. We compare this to our experience using FJ without EVAC therapy. We have had similar patients that have undergone FJ without EVAC and had a postoperative leak requiring further intervention with a stent to resolve the leak.

A chronic leak of greater than 90 days will undoubtedly have a lining to the fistula tract and cavity, making it difficult to resolve with endoscopic stent placement alone and needing some surgical intervention. In a separate study, we have shown that EVAC therapy alone can heal fistulas in our own case series of nine patients [4], but with a prolonged hospital course of mean time of healing being 50 days. If EVAC and FJ are used together, as described here, we can likely avoid prolonged healing times and hospital stays. EVAC therapy can debride the fistula cavity and tract and prepare the tissue for definitive surgical therapy with FJ, while maintaining source control. We are proposing that using these two techniques together in a chronic fistula, existing greater than 90 days, can assist in healing, disposition, and return to a diet faster than when EVAC is used alone.

There are five published reports describing FJ usage in 48 patients (Table 1) [2, 5–9]. The largest series by Chouillard et al. showed 27 patients with complete resolution of the chronic fistula; 3 patients were lost to follow-up. Vilallonga et al. showed 18 patients with chronic fistulas and a mean time of total hospital length of stay at 18.4 days [8]. The other three publications had one patient in each report, all demonstrating the feasibility and usage of FJ. Duration of healing of the fistula after FJ is reported only in the Vilallonga et al. series with a mean duration of healing after FJ at 13.5 days and a standard deviation of 10.3 days [8].

## Figures and Tables

**Table 1:** Review of fistulojejunostomy in the literature.

	*N*	Time to FJ	Open versus Lap FJ	Resolution with FJ	Hospital LOS	Days to resolution after FJ	Fail endotherapy
Baltasar et al. [7]	1	7 weeks	Open	Yes	NR	NR	Yes
Zachariah et al. [6]	1	20 days	NR	NR	NR	NR	NR
Safadi et al. [5]	1	NR	NR	Yes	NR	NR	Yes
Vilallonga et al. [8]	18	NR	NR	Yes	18.4 days	13.5 days	Yes
Chouillard et al. [2]^∗^	27	NR	27 Lap versus 3 open	Yes	NR	NR	Yes
Total	48						
P1	1	190 days	Lap	Yes	161 days	23 days	Yes
P2	1	509 days	Lap	No	34 days	74 days	Yes

^∗^Interval report of midterm results for the cohort. Three patients lost to follow-up following their surgery; Lap FJ, laparoscopic fistulojejunostomy; LOS, length of stay; NR, not reported.
